# RNA-Seq dataset of gills of the blue mussel *Mytilus* sp. infected with a low- and high-virulent strain of the mollusc pathogen *Francisella halioticida*

**DOI:** 10.1016/j.dib.2026.113399

**Published:** 2026-11-02

**Authors:** Hélène Bouras, Marie-Pierre Dubos, Maryline Houssin, Erwan Corre, Céline Zatylny-Gaudin

**Affiliations:** a University of Caen Normandy, Marine Ecosystems and oRganisms research Laboratory (MERSEA), Esplanade de la Paix, 14032 Caen, France; b LABÉO Frank Duncombe, 1 Route de Rosel, 14053 Caen Cedex 4, France; c CNRS, Sorbonne Université, FR2424, ABiMS-IFB, Station Biologique, Place Georges Teissier, Roscoff, France

**Keywords:** Mollusc pathogen, Host-pathogen interaction, Mucosal immunity, Bivalvia mollusc

## Abstract

In the scope of understanding mussel mortalities in France and the immune response of the blue mussel *Mytilus* sp. toward the emerging pathogen *Francisella halioticida*, an experimental challenge was performed. Blue mussels, *Mytilus* sp., farmed in Agon-Coutainville, Normandy, France, were infected with sterile broth (control), a low virulent (FR21) or a highly virulent (FR22c) isolate of the species *F. halioticida*. Gills samples were harvested before injection, at 24 and at 72 h post-injection, to form five pools of five mussels per condition. RNA was extracted using conventional Trizol™ protocol and mRNA were sequenced with the Illumina technology. The global transcriptome was performed *de novo* and successively mapped onto available genome sequences of *M. edulis* and *M. galloprovinciali*s, forming a total of 141 063 predicted coding sequences. The dataset can allow to distinguish distinct responses of natural hybrid mussels to two isolates with distinct virulence pattern. This dataset can be used to better understand response in the gills and can also enable the detection of novel genes of interest.

Specifications Table

**Table:** 

Subject	Biology
Specific subject area	Transcriptional response of the lophotrochozoan *Mytilus* sp. challenged with the emerging pathogen *Francisella halioticida*
Type of data	mRNA-seq Raw, Analysed, Filtered, Processed
Data collection	Adult blue mussels were injected with sterile broth, low‑virulent FR21 or high‑virulent FR22c *F. halioticida* strains. Gill samples were taken before injection and at 24 h and 72 h post‑infection (five pools of five mussels). RNA was extracted (TRIzol) and sequenced on Illumina NovaSeq (2 × 150 bp, ≈20 M PE reads). Reads were trimmed, normalized, assembled *de novo* with rnaSPAdes, annotated, quantified with Salmon and differential expression analyzed with DESeq2.
Data source location	The mussels originate from the farming area of Agon-Coutainville, Normandy France and the infected was performed at the technical center SMEL in Blainville-sur-Mer, Normandy, France. The data are stored at the laboratory MERSEA, University of Caen Normandy, France
Data accessibility	All raw and processed data are available on Sequence Read Archive (SRA) Repository name: RNA-Seq *Mytilus edulis* infected by *Francisella halioticida* Data identification number: **PRJNA1378518** [1] Direct URL to raw data: https://dataview.ncbi.nlm.nih.gov/object/PRJNA1378518?reviewer=98tegmrguu637os5j2s8et70ol Script, total transcriptome and raw count matrix: 10.5281/zenodo.22705482
Related research article	None

## 1 Value of the Data

• This study represents the first transcriptional response analysis of a marine mollusc toward a bacterial of the genus *Francisella*.
• This study focuses on the response of *Mytilus* sp. experimentally infected with two isolates showing distinct level of virulence of the shellfish pathogen
• The target of this study is the gills, an organ with increasing interest for studying mussel immunity.
• This data can be re-analyzed and might be compared with previous studies made with bacterial species of the genus *Vibrio*, where more studies are available.

## 2 Background

Mass mortality events affecting mussel populations (Mytilus spp.) have been reported in France and the Netherlands in the last decade [2,3]. In 2020, Francisella halioticida, previously described as a pathogen of abalone and scallops, was suggested as a possible causative agent [4]. Subsequent studies led to the isolation of several F. halioticida strains from mussel mortality cases, including isolates exhibiting different levels of virulence [5]. These findings highlighted the need to investigate host–pathogen interactions in mussels, a species of ecological and economic importance in European aquaculture.

Research on Francisella spp. has mostly focused on vertebrate hosts, where the pathogen is known to manipulate both innate and adaptive immunity. In contrast, mussels lack adaptive immunity and rely entirely on innate defense mechanisms, which have been characterized mainly in response to Vibrio spp. infections [6]. To date, no study has generated transcriptomic data on mussel gills exposed to F. halioticida.

The dataset presented here was produced to provide a molecular resource on the immune response of Mytilus spp. during experimental infection with two F. halioticida isolates of contrasting virulence. It complements previously published analyses by offering publicly available transcriptomic data for further exploration.

## 3 Data Description

Adult blue mussels, *Mytilus* sp. injected with either sterile broth (control), the low-virulent *F. halioticida* FR21 isolate or the high-virulent *F. halioticida* FR22c isolate were maintained in water-flow tank for 72 h and sacrificed at 24 h or 72 h post-injection. In addition, a control batch before injection was also collected. Animals were randomly pooled to produce five pools of five animals. Gills were harvested, total RNA extracted and the mRNA were sequenced with the Illumina paired-end technology. The quality of the raw sequences was assessed with FastQC and complied with MultiQC. The raw reads were processed with Trimmotatic and the quality was assessed. Raw and processed sequences in fastq are available in the Sequence Read Archive (SRA) under the accession number PRJNA1378518. Naming convention for the samples were ‘Timepoint of sacrifice (T0-T1=24h-T2=72 h)-pool replicate (P1 to P5)-BR(=gills)’. Raw sequences are followed by ‘R1’ or R2’ and processed sequences by ‘1P’ or ‘2P’. The number of sequences per samples before processing ranged from 1.9 × 10^7^ to 2.9 × 10^7^ and once processed, from 1.2 × 10^7^ to 2.0 × 10^7^. All sequences passed the mean Quality score filter with scores > 30 (Fig. 1).

**Fig. 1:**
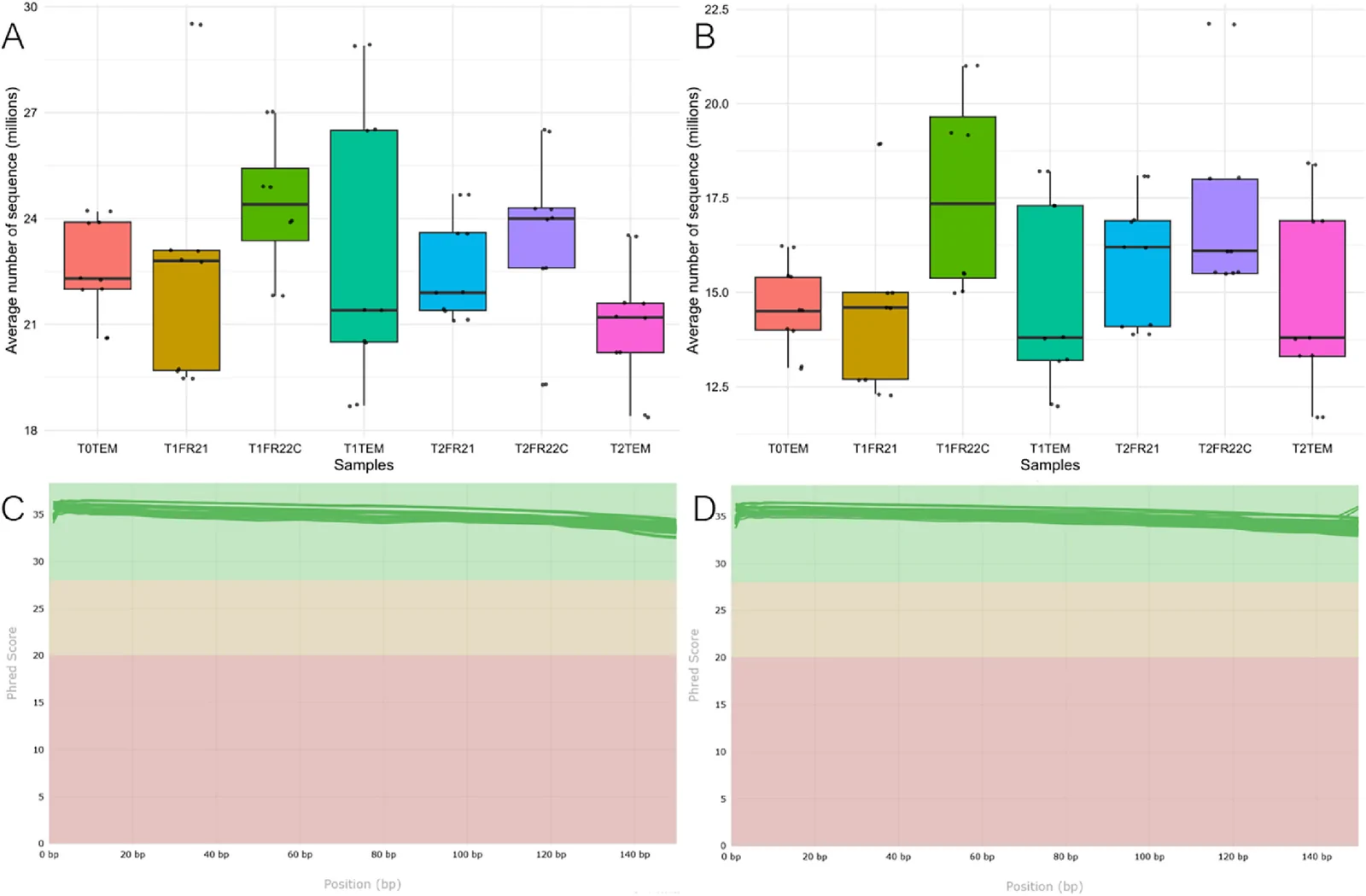
Summary of raw paired-end reads (A, C) and processed paired-end reads with Trimmomatic v0.39 (B, D) based on the number of sequences per conditions (A,B) and quality of bases (C, D).

The transcriptome was produced with a *de novo* approach using the software rnaSPAdes, producing a large transcriptome of 815 591 sequences. To improve the quality of the transcriptome, alternative post-assembly treatment was used. To reduce the transcriptome and restrict it to potential genes, it was mapped to reference genomes. In Normandy, farmed mussels are mainly natural hybrids [7]. Accordingly, the transcriptome was successively mapped on *M. edulis* MEDL1 genome (GCA_905,397,895.1) and non-mapping transcripts were then mapped onto *M. galloprovincialis* MGAL10 genome (GCA_900,618,805.1). Finally, a redundancy reduction was performed at 90% with cd-hit-est allowing to reach 401 644 sequences. The software transdecoder was used to finally predict 141 063 coding sequences. The final transcriptome has a N50 value of 1286 bp, with a median read length at 998 bp and ranging from 255 to 15 195 bp (Table 1). To assess the quality of completion of the transcriptome, a BUSCO analysis was performed, leading to a completeness of 96% in the Eukaryota database and 92.5% in the Mollusca database. However, a high level of duplication was observed with respectively 38 and 43%.

**Table 1:** Main statistics of the final transcriptome.

Number of reads	12–20 Mb/sample
Output of transcriptome (hard_filtered rnaSPAdes)	815 591
Output mapping	675 166
Output final	401 644
N50 (total)	1785
CDS	141 063
Total bases assembled	140,787,353
N50 (CDS)	1286
GC%	37.02
Average read length (bp)	998.05
Median read length (bp)	774
Range read length (bp)	255–15,195
Busco Completeness (Eukaryota / Mollusca)	96% / 92.5%
Busco Duplication (Eukaryota/ Mollusca)	38%/43%
	

To observe the transcriptional response according to the strain injected, a differential gene expression analysis was performed with DESeq2. The bio replicates ACP (Fig. 2A) and the main heatmap (Fig. 2B) leads to similar observation. A groupment of the control conditions over time and a gathering of samples at 24 h post-injection to see whether the strain is injected.

**Fig. 2:**
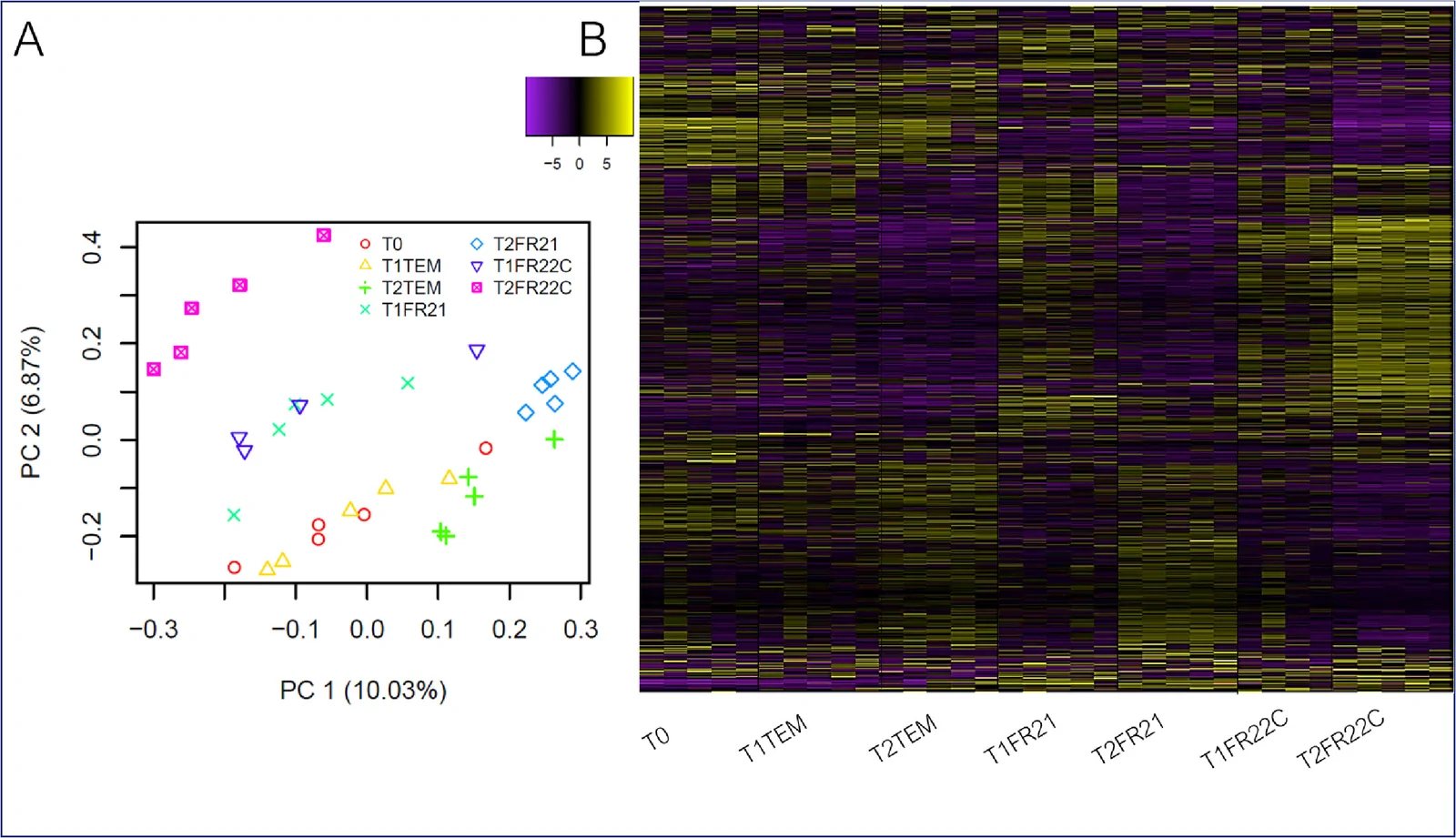
Samples expression pattern. Principal component analysis (PCA) of the different replicates based on the count matrix (A) and subsequent heatmap of expression of the different samples (B) performed using the Trinity downstream script. The samples are presented in the order of the replicates in the expression heatmap (replicate 1 to replicate 5 in each condition).

When the condition animals injected with FR21 and injected with FR22c are compared, 1052 genes are differentially expressed with 766 upregulated and 286 downregulated in animals injected with FR22C compared to those injected with FR21 (Fig. 3).

**Fig. 3:**
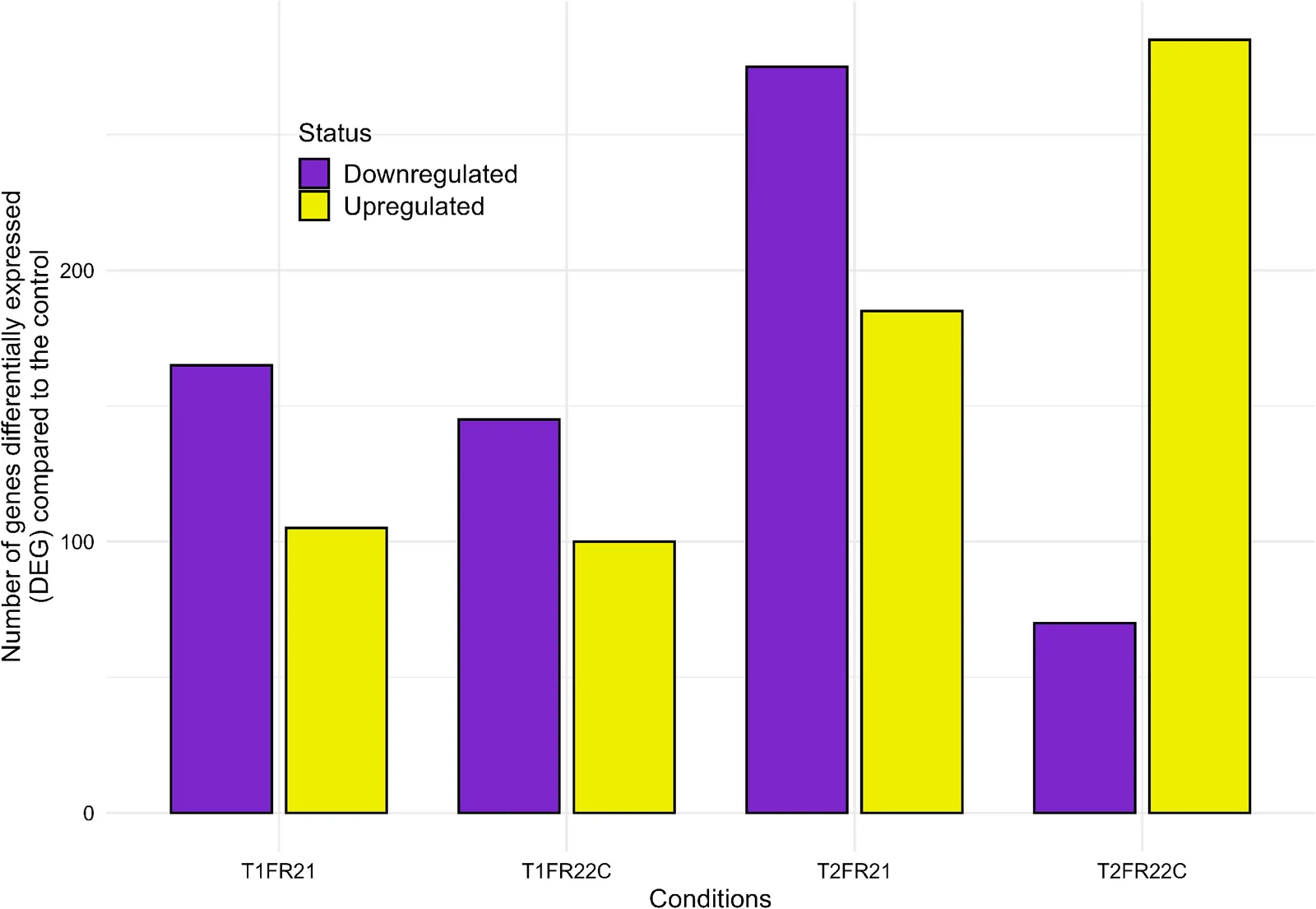
Number of genes differentially downregulated (purple) or upregulated (yellow) compared to the control conditions (T1TEM for T1 conditions and T2TEM for T2 conditions) (padj < 0.001 and abs(log2FC) > 1.2).

## 4 Experimental Design, Materials and Methods

### 4.1 Experimental design

To better understand the difference in virulence between *F. halioticida* observed in our previous study [5], adult blue mussel were injected with two strains having two virulence level (Fig. 1). In this study, the gills were analyzed through RNA-seq. Samples were harvested at two time-point, 24 h and 72 h post-injection.

### 4.2 Generation of samples

Bacterial solutions were prepared accordingly to our previous study [5]. The bacterial strains *F. halioticida* FR21 and FR22c considered as lowly virulent and highly virulent in mussels [5], respectively were cultured in modified eugon broth (MEB) supplemented with 2 and 4 mM FeCl_3_, respectively for four days at 17.5 °C with 150 rpm agitation. The broth was subcultured with a transfer of 100 µl of the FR21 culture or 500 µl of FR22c culture in MEB 2 mM FeCl_3_ for three days at 17.5 °C at 150 rpm agitation. The day of the experiment, broths were adjusted to optical density at 600 nm (OD_600nm_) 0.3. Further dilutions of 1:10 for FR21 and 1:4 for FR22c were realized to obtain the given concentration 1–5 × 10^7^ CFU/mL and solutions were used as such. Conventional plating was performed to confirmed injected concentration was 2.1 × 10^7^ CFU/mL for FR21 and 2.5 × 10^7^ CFU/mL for FR22c.

Adult farmed mussels from Agon-Coutainville, Normandy, France (January, seawater 10 °C) were harvested in large tanks and acclimated over two weeks to gently reach 15 °C. Prior to anesthesia, mussels were gently detached and anesthetized during one hour with immersion in magnesium chloride solution (0.2 M MgCl_2_·6H_2_O prepared with 30% artificial sterile seawater in ultrapure water). Once anesthetized, mussels were randomly distributed in three batches of 90 mussels and each batch was injected with either 50 µl of sterile MEB 2 mM FeCl_3_ broth, 50 µl of FR21 solution or 50 µl of FR22c solution with an insulin syringe connected to a 26 G ½ 13 mm needle. Mussels were set at room temperature (18 °C) for 15 min and each batch of 90 mussels were deposited in one single tank with sterilized seawater (filtered and UV treated) constant flux of 12 L/h per condition. Water and feeding were shared between the three tanks and distributed individual tubular system. Feeding of *Isochrysis galbana* was added 12-hour post-infection at a ratio of 1 × 10^6^ cells/day/mussel. Heavy spawning was observed within the first hour for all mussels with a gradient with mussels injected with FR22c showing the heaviest release, followed by mussels injected by FR21 and mussels injected with sterile broth. To avoid a too strong stress due to gametes presence in water, the flux was increased to 20L/h for 12 h (prior to feeding).

Samples were harvested from mussels prior to injection (control batch), at 24 h post-infection and at 72 h post-infection. For each sampling and each condition, five pools of five mussels were analyzed. This pooling strategy was set-up to reduce interindividual of each animal due to the strong presence-absence variation in *Mytilus* genus [8]. For each pool, each animal’s gills were harvested, mashed and 50 mg was dropped into a sterile Eppendorf tube to form 250 mg of gills from 5 mussels. The pool was conserved in 1 mL RNAlater at 4 °C until extraction.

### 4.3 RNA extraction

Gills were smashed in liquid nitrogen (−160 °C) and 50 mg was conserved for extraction. For each sample, extraction of total RNA was performed with TRIzol™ (Invitrogen™) according to the manufacturer protocol at the exception of 100 µl of 1‑bromo-3-chloropropane (BCP) instead of 200 µl of chloroform. Quality control check was performed with Nanodrop 2.0 and diluted before shipping to the sequencing platform.

#### 4.3.1 Illumina sequencing

Library preparation was performed at Azenta/GENEWIZ (Azenta Life Science Inc.) on the platform Illumina NovaSeq in the configuration 2 × 150 bp with 20 M paired end reads deep per sample. Raws reads were deposit in the BioProject PRJNA1378518. The global transcriptome was then created using 35 gills samples.

#### 4.3.2 *De novo* transcriptome and differential expression analysis

Raw reads were prefiltered with Trimmomatic v0.39 [9] and for each samples, the paired reads were normalized using the dedicated Trinity option [10] and then assembled using rnaSPAdes v3.15.5 [11]. As quality check, the prefiltered reads was mapped onto the final hard filtered transcriptome assembly issued from the rnaSPAdes with salmon v1.10.0 [12]. To select *Mytilus* spp. genes, the hard filtered transcriptome was mapped onto the *Mytilus edulis* MEDL1 genome (GCA_905,397,895.1) using minimap2 v2.25 [13] and the unmapped reads were then mapped to *Mytilus galloprovincialis* MGAL10 genome (GCA_900,618,805.1). The merged file from those two mappings were clustered using cd-hit-est at 90% treshold [14,15] to reduce as possible the redundancy. Finally, the coding sequences were predicted with TransDecoder v5.7.1. As a final quality check, the obtained transcriptome was verified by remapping the filtered reads against it using salmon [12]. Assembly quality was assessed with BUSCO [16]. Bio replicates analyses was performed with PtR script available as companion with Trinity [17]. Global transcriptome was annotated with eggnog-mapper 2.1.8 with eggnog Database 5.0.2 [18,19] on Galaxy server [20]. The quantification of transcripts for each sample was performed with Salmon using default parameters [12] and differential expression (DE) analysis with DESeq2 using default parameters [21].

All parameters for each command line can be found at this GitHub Repository: https://github.com/hb660/DataPaper_RNASEQ/blob/main/README.md

## Limitations

The dataset was generated from naturally occurring hybrid mussels, resulting in high genomic heterogeneity among individuals. Because no haplotype-resolved reference genome is currently available for these hybrids, transcript reconstruction was performed using a *de novo* assembly strategy. The dataset therefore contains potential transcript redundancy associated with allelic variants and expanded gene family’s characteristic of *Mytilus* genomes. Presence–absence variation (PAV), commonly reported in mussels, may also contribute to inter-individual variability within the dataset.

All specimens were actively spawning at the onset of the experiment. Differences in spawning intensity among individuals and treatment groups were observed during the early phase of sampling (from low in control, medium when injected with FR21 and high with FR22c). It is to be noted that water flow was increased to limit gamete accumulation.

## Ethics Statement

The authors state that our study on aquatic invertebrates do not refer to the objectives of the “EU Directive 2010/63/EU on the protection of animals used for scientific purposes”, based on Article 1(3). The authors have read and follow the ethical requirements for publication in Data in Brief and confirm that the current work does not involve human subjects, animal experiments under restriction, or any data collected from social media platforms.

## CRediT Author Statement

**Helene Bouras:** Conceptualization (equal); Methodology (lead); Validation (equal); Formal analysis (lead); Investigation (lead); Data Curation (lead); Visualization (lead); Writing – Original Draft (lead). **Marie-Pierre Dubos**: Methodology (supporting); Formal analysis (supporting). **Maryline Houssin:** Conceptualization (equal); Methodology (supporting); Project administration (supporting), Funding acquisition (supporting), Supervision (equal), **Erwan Corre**: Methodology (supporting); Investigation (supporting); Validation (equal); Writing – Review & Editing (equal). **Celine Zatylny-Gaudin:** Conceptualization (equal); Methodology (supporting); Validation (equal); Project administration (lead), Funding acquisition (supporting), Supervision (equal), Writing – Review & Editing (equal).

## Data Availability

sraRNA-Seq Mytilus edulis infected by Francisella halioticida (Original data).
